# Pharmacokinetics of Natural and Engineered Secreted Factors Delivered by Mesenchymal Stromal Cells

**DOI:** 10.1371/journal.pone.0089882

**Published:** 2014-02-21

**Authors:** Jessica S. Elman, Ryan M. Murray, Fangjing Wang, Keyue Shen, Shan Gao, Kevin E. Conway, Martin L. Yarmush, Bakhos A. Tannous, Ralph Weissleder, Biju Parekkadan

**Affiliations:** 1 Department of Surgery, Center for Engineering in Medicine and Surgical Services, Massachusetts General Hospital, Harvard Medical School and the Shriners Hospital for Children, Boston, Massachusetts, United States of America; 2 Department of Biomedical Engineering, Rutgers University, Piscataway, New Jersey, United States of America; 3 Center for Systems Biology, Massachusetts General Hospital, Harvard Medical School, Boston, Massachusetts, United States of America; 4 Department of Neurology, Experimental Therapeutics and Molecular Imaging Laboratory, Massachusetts General Hospital, Charlestown, Massachusetts, United States of America; 5 Harvard Stem Cell Institute, Boston, Massachusetts, United States of America; National Institutes of Health, United States of America

## Abstract

Transient cell therapy is an emerging drug class that requires new approaches for pharmacological monitoring during use. Human mesenchymal stem cells (MSCs) are a clinically-tested transient cell therapeutic that naturally secrete anti-inflammatory factors to attenuate immune-mediated diseases. MSCs were used as a proof-of-concept with the hypothesis that measuring the release of secreted factors after cell transplantation, rather than the biodistribution of the cells alone, would be an alternative monitoring tool to understand the exposure of a subject to MSCs. By comparing cellular engraftment and the associated serum concentration of secreted factors released from the graft, we observed clear differences between the pharmacokinetics of MSCs and their secreted factors. Exploration of the effects of natural or engineered secreted proteins, active cellular secretion pathways, and clearance mechanisms revealed novel aspects that affect the systemic exposure of the host to secreted factors from a cellular therapeutic. We assert that a combined consideration of cell delivery strategies and molecular pharmacokinetics can provide a more predictive model for outcomes of MSC transplantation and potentially other transient cell therapeutics.

## Introduction

Cell therapy is an exponentially growing field with >2,500 clinical trials in the world over the last 10 years [1]. Cell-based therapeutic products are positioned as a billion dollar per year industry with anticipated market growth [2], [3]. The method of using cells as drugs is particularly advantageous when a higher-order approach to treatment is required. A fundamental issue, particularly for transient cell therapies which are the largest of the drug class (50%), is the lack of predictive measures of the body’s response to the therapy and vice versa: traditionally known as a drug’s pharmacokinetics (PK) and pharmacodynamics (PD). Without a rigorous PK/PD model of the mechanism of action of transient cell therapies, processes cannot be optimized to formulate a drug and physicians will be unable to effectively monitor and communicate the benefits and risks of a cell therapy to a patient.

Mesenchymal stem cells (MSCs) - an adult multipotent progenitor cell population initially derived from bone marrow [4] – have over a decade of clinical testing in thousands of patients worldwide. These cells have appealing manufacturing properties such as the ease of isolation, expansion, and cryopreservation, as well as the tolerability of allogeneic cell therapy. MSCs are widely being evaluated for the combinatorial treatment of a variety of diseases including myocardial infarction [5], [6], bone marrow transplantation [7], stroke [8], autoimmune disease [9], and wound healing [10], [11], [12], [13]. The predominant use of MSCs as an immunomodulatory agent is substantiated by the observation that MSCs can inhibit the activation and effector function of numerous immune cell types [14], [15], [16], [17]. The mechanism of action of immune cell inhibition by MSCs is primarily due to the release of secreted factors by cells [18]. MSCs have proven effective in early stage clinical trials [19], yet, several Phase II and Phase III industry-led trials either have undergone early termination or have failed to meet primary endpoints [20]. New strategies to optimize this therapeutic are needed to help usher this promising cell population to the clinic.

A deep understanding of MSC pharmacology can provide insight on how to best deliver this therapeutic. A prevailing view of MSC trafficking is that, upon intravenous administration, cell homing, engraftment, proliferation, and/or differentiation are critical to induce a therapeutic effect. Sensitive monitoring techniques have demonstrated little, if any, long-term engraftment (*>*1 week) of MSCs upon systemic administration with the majority of administered MSCs (*>*90%) accumulating immediately in the lungs and then cleared with a half-life of 24 h [21], [22], [23]. Therefore, MSCs can be viewed conceptually as a transient cell therapy that delivers a “payload” of secreted factors to alter acute disease progression [20].

We put the concept of MSCs as a therapeutic delivery vehicle to the test with a focus on evaluating the systemic release of secreted factors. Investigators have studied the release of natural secreted proteins such as TSG-6 [22] and TRAIL [24] or engineered antibodies [25] from MSCs in the systemic circulation after transplantation, although a thorough pharmacological analysis was not performed nor compared to purified proteins. In this study, conventional cell biodistribution data was combined with serum profiles of natural or engineered secreted factors released by the cells to understand rate-limiting processes that alter exposure to intravenous MSC therapy, the most widely used administration method. Based on our combined PK analysis, we observed that molecular monitoring of serum secreted factors revealed interesting phenomena regarding the delivery of natural or engineered proteins, an active cell secretion mechanism, and the host immune response to the graft. This study can aid in designing an optimal cell delivery regimen to maximize MSC therapy.

## Results

### Comparison between the Bioavailability of Transplanted MSCs and Secreted Molecules

**Figure 1:**
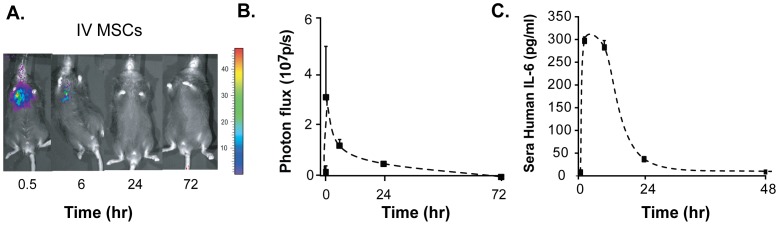
Combined pharmacokinetic monitoring of MSCs and MSC-secreted IL-6. (A) Bioluminescent images of C57Bl/6 mice over a period of three days after IV cell administration of one million luciferase-engineered human MSCs. (B) Photon flux of bioluminescent signal over time after IV cell administration. Durable BLI signals were detected up to 24 hours in mice that were injected IV with MSCs. (C) Serum ELISA measurements of human IL-6 released by IV cell transplants over time. Time points for serum and imaging analyses were 0.5, 8, 24, and 72 hours after cell injection. Pooled mouse serum was serially analyzed as batches of N = 5.

Secreted factors released by MSCs after transplantation have been a general phenomena that has been observed in several pre-clinical therapeutic studies [22], [26]. It is reported that MSCs can secrete a wide range of cytokines and growth factors [18]. We designed a pharmacokinetic study to monitor both the viability and distribution of MSC transplants and overlay the serum profiles of a specific and detectable level of interleukin (IL)-6, a pleiotropic molecule secreted by MSCs [27]. Human MSCs were engineered with a luciferase reporter gene, injected via intravenous (IV) route (the convention used by the cell therapy community) and detected after injection using whole-body, bioluminescent imaging (BLI). By nearly 8 hours after IV cell injection the BLI signal was almost undetectable (**Figure 1A**). At the same imaging time points, using human MSCs that were not engineered, we also measured the serum levels of human IL-6. We observed that the release of human IL-6 by MSCs was short-lived and tracked accordingly to the cellular BLI signal of the graft (**Figure 1C**). Similar pharmacokinetics of two other protein secreted factors, monocyte chemoattrive protein (MCP)-1 and IL-8, were observed after IV injection in vivo further supporting our observation (**Figure S2A–B**). Future experiments continued to explore and evaluate the impact of these pharmacokinetic profiles, with a focus on molecular delivery of IL-6 by IV administration.

### Cell Delivery of IL-6 Achieves Greater Exposure than Molecular Delivery of IL-6

**Figure 2:**
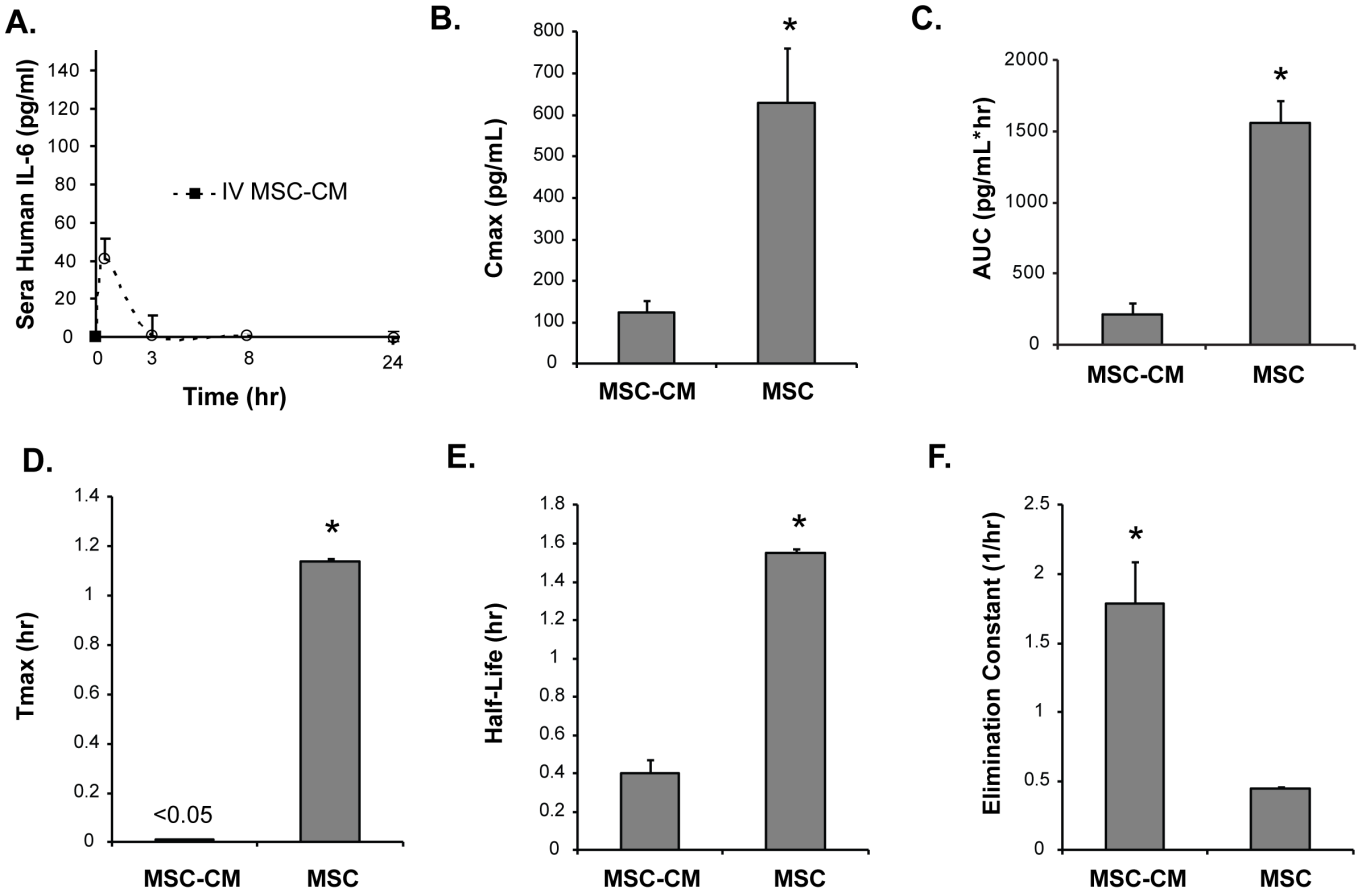
Enhanced delivery of IL-6 by MSC transplants compared to MSC conditioned medium. (A) Serum profiles of human IL-6 after IV administration of concentrated conditioned medium into C57Bl/6 mice. The plot was normalized to the dose of conditioned medium that was contributed by 1×10^6^ cells. Pharmacokinetic parameters (B) Cmax, (C) AUC, (D) Tmax, (E) Half-life, and (F) Elimination constant were calculated for IL-6 exposure by cell transplants compared to CM administration. Significant differences between cell transplants compared to CM whereby higher levels of IL-6 and longer artificial duration was observed in plasma after cell transplantation. Time points for serum analyses were 0.5, 8, and 24 hours after cell or media injection. Mice were serially analyzed as batches of N = 5 per group. * denotes P>0.01.

Cells can be considered a “carrier” for their secreted factors. We used this perspective to understand the differences of delivering IL-6 in a purified form compared to a cell transplant. Conditioned medium from MSCs (referred to as MSC-CM) was generated in order to compare MSC-derived IL-6 without concerns of post-translational modifications that take place in other protein expression systems for recombinant forms of IL-6 that may affect bioavailability. Concentrated MSC-CM was prepared and a volume of 400 µL (containing 40 ng IL-6) was injected into mice. The content of soluble factors in this volume is equivalent to the secreted levels when 3×10^6^ cells are cultured for 2 days in vitro and was normalized to 1×10^6^ cells for comparison to cell transplants at the same cell dose. MSC-CM was administered by IV and the serum levels of IL-6 followed a classical bolus pharmacokinetic profile (**Figure 2A**). Pharmacokinetic parameters for serum IL-6 levels were calculated with the assumption that the clearance of IL-6 itself was a constant 218 ml/hr based on the literature [28]. The maximum serum IL-6 concentration was increased by ∼400% when delivered by cell transplants (**Figure 2B**). This amounted to a greater than 7-fold increase in the exposure of the subject to IL-6 as measured by area under the curve (AUC) analysis (**Figure 2C**). The temporal kinetics of IL-6 was also artificially extended by way of cell transplantation. The time to reach maximum serum concentration, the half-life, and the elimination constant of IL-6 were all significantly modified for prolonged duration of IL-6 by the use of cell transplantation (**Figure 2D–F**). These data highlight the supplementary changes to molecular pharmacokinetic parameters by way of cellular delivery.

### MSCs Utilize an Active, Golgi-Dependent Secretion Mechnaism to Release IL-6 *In Vivo*

**Figure 3:**
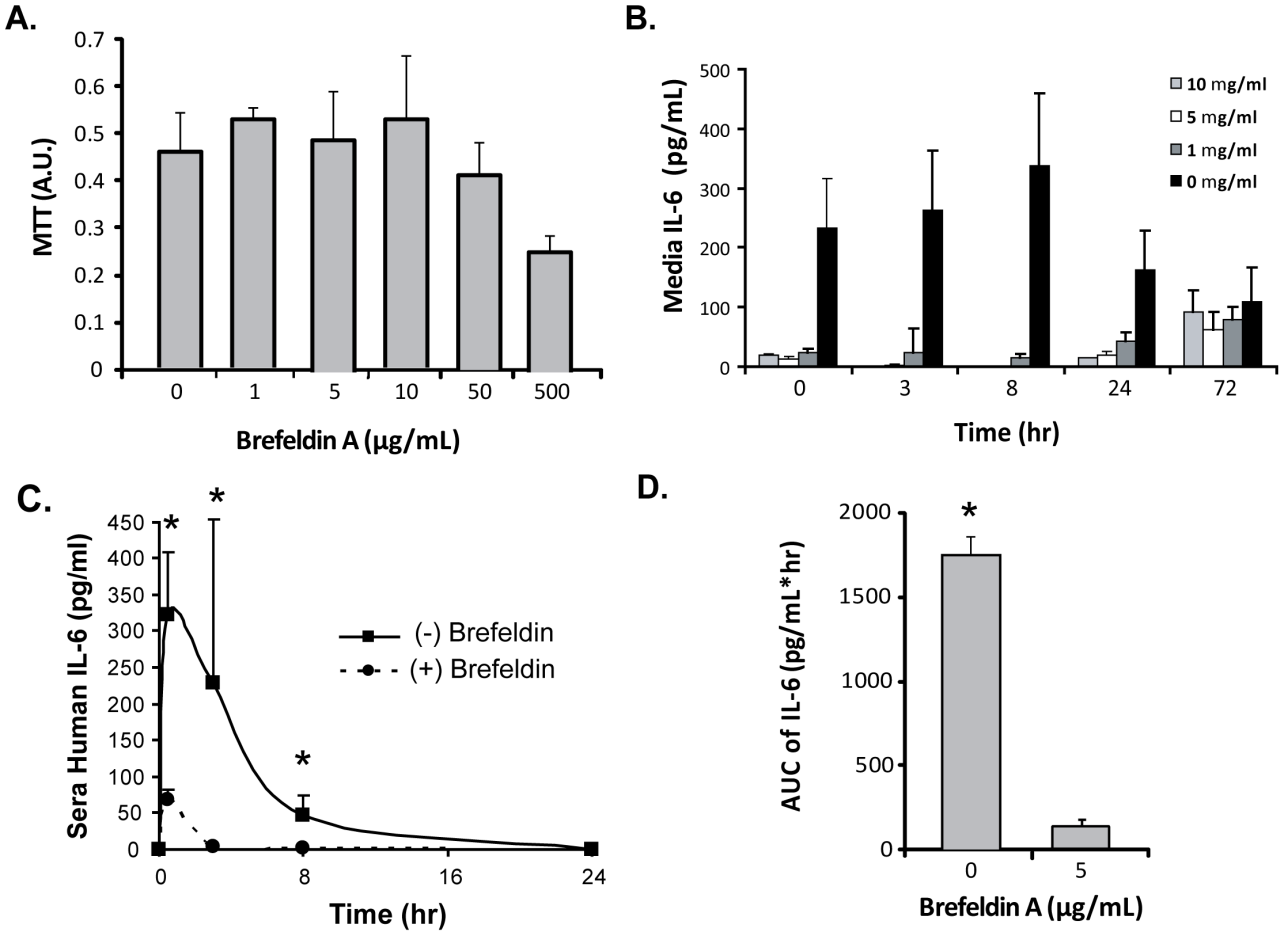
Golgi-dependent secretion mechanism of MSC-derived IL-6 in vivo. Brefeldin A pre-treatment of MSCs was used to evaluate blockade of IL-6 release in vitro and in vivo. (A) MTT assay of MSCs treated at different concentrations of brefeldin. A non-toxic dose of 5 ug/ml was used for functional studies. (B) Human IL-6 levels in vitro after brefeldin pre-treatment. Significant reduction in 24 hour release of IL-6 was observed across all doses. (C) Alteration in serum IL-6 delivery by MSCs pretreated with a Golgi-apparatus inhibitor, Brefeldin A. MSCs were incubated with 5 µg/ml of BFA for one day and then injected into C57Bl/6 mice and compared to untreated MSCs in terms of serum IL-6 delivery. Brefeldin treatment of MSCs led to diminished release of human IL-6 in vitro and in vivo. (D) Area-under-curve analysis of human IL-6 after MSC pre-treatment with brefeldin A and transplantation. Exposure to IL-6 was significantly reduced by inhibition of the Golgi apparatus. Time points for serum analyses were 0.5, 8, and 24 hours after cell injection. Mice were serially analyzed as batches of N = 5 per group. * denotes P>0.01.

The presence of human IL-6 in serum after cell transplantation could be due to passive mechanisms, such as cell rupture and cytokine release, or by active secretion pathways. Brefeldin A (BFA), a protein-transport inhibitor, was used to block IL-6 production by inhibiting Golgi apparatus-dependent vesicle secretion. A non-toxic concentration of 5 ug/ml BFA was chosen by evaluating a dose response of BFA to MSCs in vitro (**Figure 3A**). After incubating MSCs with BFA for one day, the in vitro secretion of IL-6 in the supernatant was significantly inhibited compared to the untreated controls (**Figure 3B**). The secretion of IL-6 was not restored after a day of incubation with BFA until 72 hrs later (**Figure 3B**). We were satisfied with blockade of IL-6 for >60 hours in vitro and advanced this cell formulation with impaired IL-6 secretion for in vivo PK studies. The PK profile of MSC-derived IL-6 was dramatically different, specifically a reduced maximal effective concentration (∼5×) and AUC (∼1000×) compared to the cells that were not treated with BFA (**Figure 3C–D**). This study highlights a powerful active mechanism that MSCs employ to deliver IL-6 to the bloodstream that requires the Golgi apparatus.

### Exposure of MSC-derived IL-6 is a Function of Host Immune Cells

**Figure 4:**
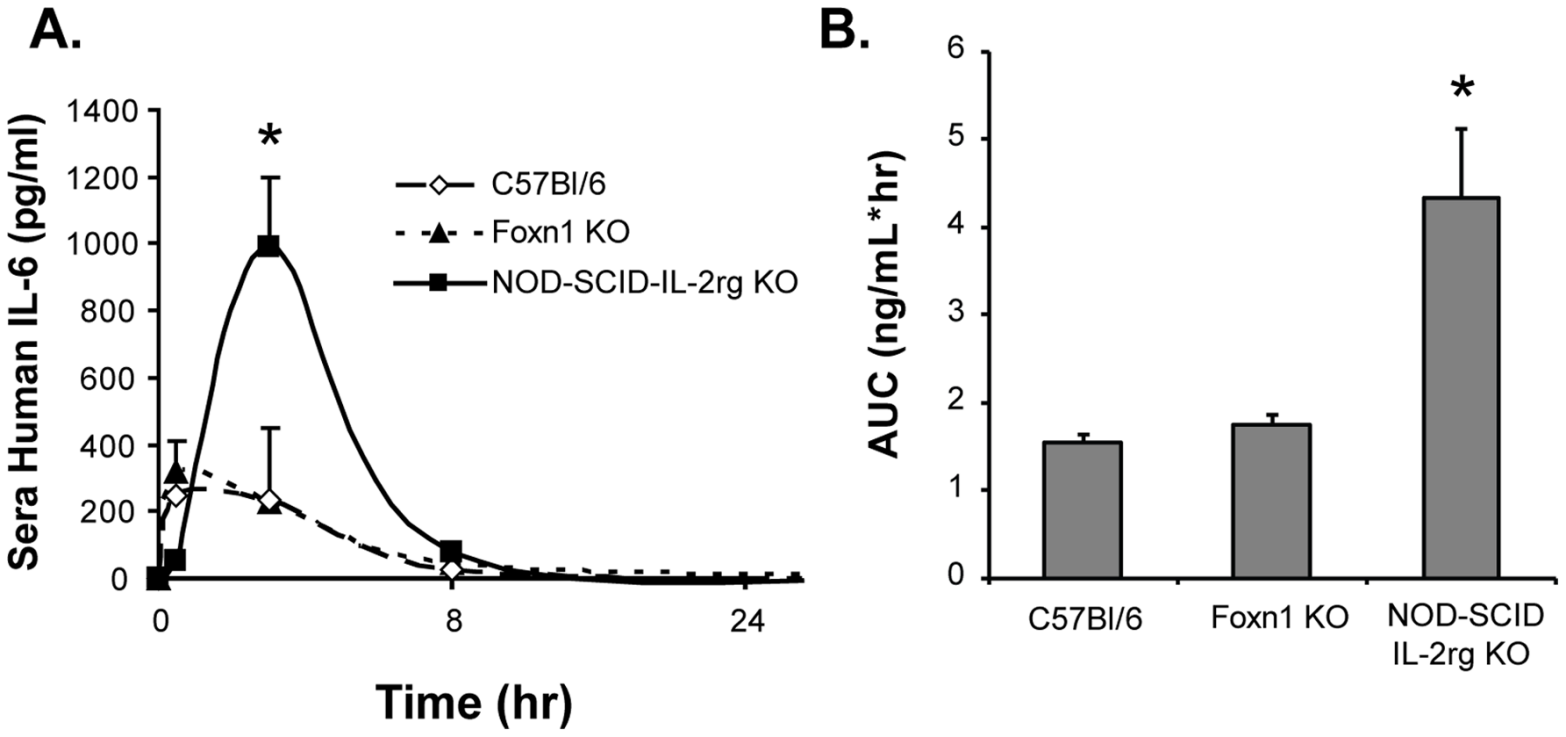
The immune system limits the bioavailability of MSC-derived IL-6. Pharmacokinetic profile of sera IL-6 after IV cell administration. Approximately 1×10^6^ MSCs were injected into C57Bl/6, Foxn1−/−, or NSG mice by IV injection. At different time points after cell injection, mice were sampled for blood plasma and serum human IL-6 levels were measured by ELISA. (B) AUC analysis of IL-6 exposure as a function of mouse strain. IV administration was significantly affected by mouse strain, particularly in severely immunodeficient mice, which had the highest exposure of IL-6 for a given cell mass. Data represent mean ± standard derivation of duplicate or triplicate experiments. Time points for serum analyses were 0.5, 8, and 24 hours after cell injection. Mice were serially analyzed as batches of N = 5 per study. * denotes P>0.01.

To study the natural process of protein release from non-engineered cells, we designed our PK model using human cell transplants in mice. This model afforded us the ability to detect human proteins in mouse serum with high specificity and sensitivity and correlate that to *in situ* cellular production. We were also interested in studying the immune response, albeit a xenogenic rejection response, and its ability to alter in vivo protein release. We transplanted human MSCs by IV administration in mice strains that are immunocompetent (C57Bl/6), less immunocompetent (Foxn1−/−, thymic and peripheral loss of T cells), or severely immunodeficient (NOD-SCID-IL-2rg −/−, loss of B, T, and NK cells). The effect of the immune response on MSC-derived IL-6 serum kinetics was pronounced after IV administration. NOD-SCID-IL-2rg −/− mice treated with human MSCs had a delayed maximum effective concentration and a longer half-life of serum IL-6 than the same data gathered from Foxn1−/−, or C57Bl/6 (**Figure 4A**). The exposure of the subject to MSC-derived IL-6 was quantifiably different amongst recipients (**Figure 4B**), suggesting that molecular monitoring of cell therapy can distinguish clearance mechanisms of the MSC transplant and/or secreted factors, in particular a form of immune clearance.

### Enhanced Systemic Exposure of Secreted Factors by Engineered MSCs

**Figure 5:**
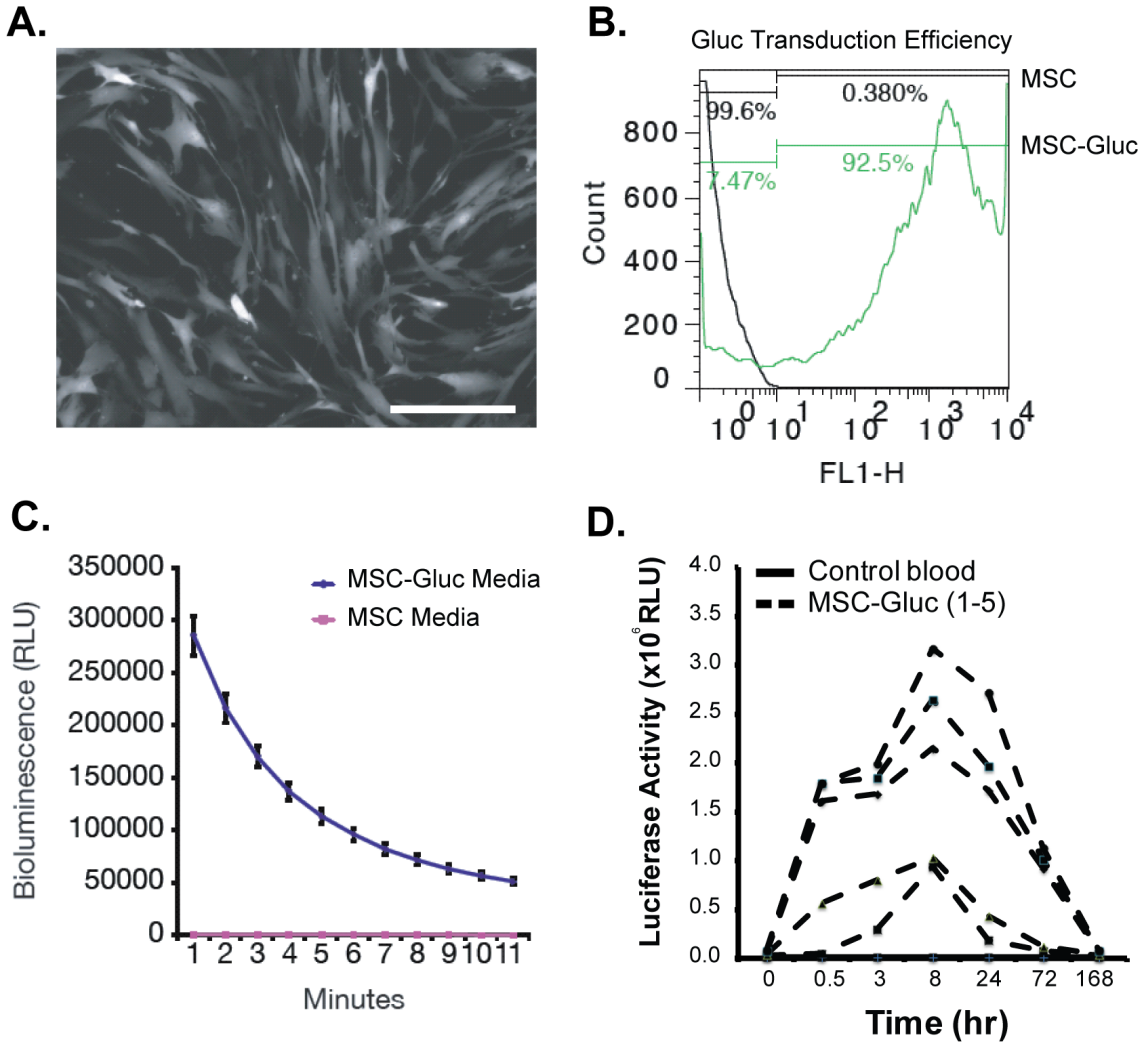
Blood Monitoring of Engineered Human MSCs with the Secreted Gaussia Luciferase Reporter. A lentivirus vector expressing GLuc and GFP was transduced into human MSCs at a confluence of 70% and multiplicity of infection of 4∶1 in complex with 8 ug/ml of polybrene. (A) GFP micrograph and (B) flow cytometry showing high expression level and therefore transduction efficiency of construct. (C) The activity of GLuc was successfully measured in MSCs conditioned medium using a luminometer. (D) Five different engineered cell lines were infused into NOD-SCID mice and serum was individually collected at 0.5, 8, 24, 72, and 168 hours after cell injection in batches of N = 5 per study. MSCs constitutively expressing GLuc were detected in many cases over a week in duration.

MSC-derived IL-6 is a natural and highly expressed secreted factor that enabled our molecular monitoring approach, however IL-6 can be influenced by the *in vivo* microenvironment that MSCs encounter. In order to understand the maximal exposure of a subject to MSC secreted factor that was uninfluenced by host regulation, we developed genetically engineered MSCs with constitutive expression of the naturally secreted Gaussia luciferase (Gluc) reporter. Gluc has a circulation half-life of 5–10 minutes in mice, and has been used as a highly sensitive reporter (detection of ∼1000 cells) for quantitative assessment of cells *in vivo* by measuring its level in the blood ex vivo [29]. GLuc activity can be easily quantified in blood by adding its substrate coelenterazine and measuring emitted photons using a luminometer. MSCs were first transduced with a lentivirus vector to stably express Gluc and GFP under the control of the constitutively active CMV promoter which yielded high transduction efficiency as monitored by fluorescent microscopy and flow cytometry for GFP (**Figure 5A–B**). Gluc expression and secretion was also readily detected in MSCs conditioned medium (**Figure 5C**). When infused into NOD-SCID mice, GLuc was detectable over a one week period with a time to peak concentration occurring at ∼8 hours post-cells transplantation (**Figure 5D**). Engineering of MSCs with GLuc revealed a longer exposure to cell therapy and suggests engineered cell formulations for therapeutic studies may be useful for minimizing cell dose and/or frequency to achieve durable responses.

## Discussion

Pharmacology is a powerful discipline with many available methods to help understand and shape the characteristics of a drug to meet a therapeutic need. Monitoring a drug’s PK is a necessary study of a drug formulation that has been modeled extensively by balancing the infusion, absorption, metabolism, and elimination of a drug, assuming the body is defined by a steady-state of discrete compartments that are permeable to a drug. Drug formulations that contain living cells are an ever-increasing class of therapeutics that can benefit from such pharmacological analysis. The classical monitoring strategy for a cell therapy is to define the trafficking and viability of a cellular formulation after introduction to the body. In this study, we coupled this strategy with molecular monitoring of secreted factors released by a cell transplant that became detectable in the systemic circulation. We identified that these two PK profiles - that is, the PK of an administered MSC population and MSC-secreted IL-6 - were discordant. This was dependent on the imaging modality used in this study (BLI), which has known limitations in whole-animal reporting due to tissue diffusion distance of light emission. BLI showed a prominent lung expression of the MSC graft for a period of several hours, which engineered MSC-Gluc releasing a secreted reporter could be detected for days. Exploration of the dynamics and rate-limiting aspects of MSC-derived secreted factors in blood serum, rather than the cells themselves, may provide more predictive power in their use as a therapeutic.

The PK of cell-derived molecules can be considered a lumped model of classical drug PK with additional complexity to consider due to cellular delivery. Cells as a delivery vehicle may be responsible for the difference in profiles compared to isolated molecules. Administration of a molecular drug bolus typically follows an exponentially decaying trajectory in terms of serum concentration of the drug. After cell administration, we observed that serum levels of secreted IL-6 followed an exponential decay independent of administration route. This may be attributed to limited engraftment MSCs entrapped in the lung. The diffusion of IL-6 from the cells would subsequently follow and be limited by tissue transport barriers before being detected in the bloodstream. In the systemic circulation secreted factors would likely follow their natural clearance pathways, which, in the case of IL-6, are very rapid (∼minutes in half-life). We propose that traditional PK models can be modified to account for an active, cellular production term as well as the diffusion of material from an engrafted tissue bed.

The elimination, or clearance, of a drug compound can occur by metabolism or excretion of the drug in voided volume. In the case of a MSC transplant, we could detect the influence that the immune system has on the exposure of a subject to MSC secreted factors. The immune response we observed is likely a xenogenic rejection response that can be compartmentalized by arms of innate and adaptive immunity. Previous studies have shown that the immune competency of the host can have an impact on the cellular viability after transplantation [30]. There are a number of reports that describe the role of NK cells and other mechanisms that affect the viability of allogeneic MSCs after transplantation including the generation of immunological memory to the transplanted material over time [16], [31], [32]. The clearance of MSCs was substantial in IV transplanted MSCs, which were entrapped in lung tissue. NOD-SCID-IL-2rg −/− mice had a much greater exposure to MSC-derived factors compared to Foxn1−/− mice or C57Bl/6 mice. The difference between the two immunocompromised strains lies in the absence of B cells, NK cells, and defects in cytokine signaling in NOD-SCID-IL-2rg −/− mice suggesting that these cell populations and cytokines may be accountable for MSC clearance. This study, however, cannot distinguish immunologic elimination of the human cells versus elimination of the human secreted protein without further evaluation.

We, and others, have observed protection of acute liver injury by molecules derived from MSCs [33], [34]. MSC molecules collectively had a dose-dependent effect on injured hepatocytes and stimulated a proliferation response in vitro and in vivo [33]. In this study, we used IL-6 as a model secreted factor to study the cellular release profile of this cytokine in vivo. Although specific mediators of therapy still remain elusive, there is considerable evidence that IL-6, itself, may be liver-protective. [35]. IL-6 is also shown to be important in improving acute inflammatory response [36], [37]. We envision that other cytokine and growth factors released by MSCs will help broaden the overall PK profile of this transient cell therapy and begin to identify associations with relevant diseases that can be combated through the combination of factors released by MSCs. Cognate receptors for specific secreted factors will help clarify the pharmacodynamics of MSC action and if dosing is enough to activate downstream signaling of MSCs are amenable to *ex vivo* engineering to express therapeutic secreted factors such as IL-2 for cancer immunotherapy [38]. In this study, we first report the use of a non-specific GLuc reporter that was engineered into MSCs for sensitive blood detection. MSC-Gluc revealed a longer exposure of the subject to a secreted factor implying that MSCs were persistent in the body although undetected by BLI. MSCs were genetically engineered ex vivo with a self-inactivating lentivirus vector which integrates the Gluc cDNA within the genome of the cells, leading to stable expression. This study cannot rule out the possibility that MSCs could fuse with a host cell (e.g. myeloid cells) after transplantation and thereby maintain a longer serum level. These data also suggest that constitutive secreted factors are necessary to reveal the true bioavailability of MSCs, as IL-6 and presumably other natural secreted factors may be regulated at the gene expression level by the host. The initial release dynamics of IL-6 were contributed by an active Golgi-dependent mechanism, which is presumed to be necessary for continued secretion of other protein factors such as GLuc. Non-protein secreted factors, such as nitric oxide or prostaglandin E2, may not be rate-limited by Golgi-secretion pathways but instead follow enzymatic reaction kinetics that require stimulation to generate these mediators *in vivo* [*14]*, *[39*]. MSC-Gluc can be an extremely useful tool for cell pharmacology studies that evaluate administration routes, initial dosage, and dosing frequency for optimizing the exposure of a cell therapy product.

This work serves as the first application of a combined cell biodistribution and molecular PK modeling approach to MSC therapeutics. By combined *in silico* modeling and empirical analysis, a functional PK/PD model can now begin to be developed to predict the nature of MSC therapy given a particular formulation and administration route. Allometric scaling laws that help predict the conversion of parameters from animal to human models may be applicable to guide clinical trials using MSC therapeutics. Although we focus on a few key mediators, a more comprehensive view of all bioactive MSC secreted factors can lead to second-generation models that better capture potential non-linearity in the data. In addition, this theoretical framework may serve as the foundation for other clinically used cell variants such as hematopoietic and embryonic stem cells, or T cells. Such predictive, *in silico* analysis of cell-based therapies may reduce experimental costs due to a systematic minimization of required testing, increase throughput of discovery, and ultimately lead to more efficacious treatment regimens.

## Materials and Methods

### Mice

Athymic Foxn1−/− mice (nude, male, 6–8 weeks old), C57BL/6 mice (male, 8 weeks old), Balbc/J mice (female, 8 weeks old), and NOD.Cg-**Prkdc^scid^ Il2rg^tm1Wjl^**/SzJ (NSG mice, male, 8–10 weeks old) were all purchased from Jackson Laboratories (Bar Harbor, ME) and housed at Massachusetts General Hospital Animal Facility following approved experimental protocols by the IACUC.

### Human MSC Isolation and Expansion

Human MSCs were isolated and expanded following a previously established protocol [33], [34]. Briefly, fresh human bone marrow aspirates were purchased from Lonza. Mononuclear cells were separated by Ficoll density gradient centrifugation (GE Healthcare) and plated on a T-175 flask (1×10^6^ cells per flask). Mononuclear cells were cultured at 37°C with 10% CO_2_ in MSC expansion medium. MSC expansion medium was composed of 15% fetal bovine serum, 2% penicillin and streptomycin, 0.2% gentamycin, 1 ng/L fibroblast growth factor, alpha-MEM with ribonucleosides and deoxyribonucleosides. Medium was changed 1 week later and unbound cells were washed away. The following week, colony-forming adherent cells were re-plated into a new flask for expansion. Medium was changed every 3–4 days. MSCs were subcultured when they reached 70–80% confluence. Only passage 2–5 MSCs were used for experiments. **Figure S1** outlines the immunophenotype of MSCs using antibodies purchased from BD Biosciences.

### MSC Administration and Measurement of Human IL-6, MCP-1, and IL-8 Levels in Plasma

MSCs (1×10^6^ MSCs in 200 µl FBS-free medium) were injected into mice by IV infusion. At 30 minutes, 3 hours, 8 hours, 24 hours and 72 hours, mice were anesthetized with 60 µl ketamine, 30 µl xylazine, and 60 µl saline per mouse and blood was withdrawn by cardiac puncture. After centrifugation at 14,000 rpm for 10 minutes at 4°C, plasma were collected and stored at −80°C before use. Human IL-6, MCP-1, and IL-8 levels were measured using an ELISA kit from BD Bioscience following the supplier’s recommended procedures.

### Preparation of Human MSCs Expressing a Firefly Luciferase Gene Reporter

The lentiviral vector pHR’MND-LRT containing a firefly luciferase reporter was constructed as previously described [40]. Infectious virus was produced by triple transient co-transduction of 293T/17 cells (ATCC) with pHR’MND-LRT, pCMVΔR8.91 i.e. packaging vector, and pMD.G i.e. VSVG pseudotyping vector. The titer of virus was determined by transduction of 293T cells followed by flow cytometry analysis of the mRFP reporter (Ex: 594 nm/Em: 620±15 nm). Cultures of 30–40% confluent human MSCs in a T-175 flask were incubated with the virus at a multiplicity of infection of 4 in a total of 20 ml expansion medium containing 8 µg/ml polybrene. This transduction protocol was repeated one more time. In each round, cells were incubated with the viral supernatant for 8 hours and then in MSC expansion medium for 16 hours. After the second round of infection, fresh medium was added to each flask and cultured for 3–4 days. Luciferase activity of transduced MSCs was confirmed with a luciferase activity assay before in vivo use.

### Bioluminescence Imaging

A total of 1×10^6^ luciferase-engineered MSCs were given to C57Bl/6 mice either IM or IV. At specific time points after cell injection, mice received an intraperitoneal injection of 4.5 mg of luciferase substrate solution (Molecular Imaging Products) and were imaged thereafter. The bioluminescent signal was measured in anesthetized mice on an IVIS-100 imaging system (Caliper LifeSciences) until a peak signal was reached. Data are expressed as photons/second/cm^2^, encompassing a region of interest over the implanted cells, including lung, leg and whole body.

### Preparation of Concentrated Conditioned Medium

MSCs were cultured to 70–80% confluency in T-175 culture flasks before 15 ml DMEM media consisted of 0.05% BSA and 2% penicillin and streptomycin were added. Cells were further cultured for 1–2 days and then the supernatants were collected and filtered. Cell number was quantified using a hemacytometer after trypsinization. Culture media were concentrated 20–50 folds using an Amicon filter (MWCO: 3,000 Da) by centrifuging at 3500 rpm for 2–3 hours. The human IL-6 levels were measured by ELISA by appropriate serial dilution before injection into mice. Concentrated conditioned medium (400 µl) were injected into C57Bl/6 mice by IV or IM routes.

### Engineering and *In Vivo* Monitoring of MSCs with Secreted Gaussia Luciferase

MSCs were allowed to grow up to about 70% confluency before viral transduction. A lentivirus vector carrying the expression cassette for Gluc and GFP, separated by an internal ribosomal entry site, under the control of the CMV promoter was previously described [29] with a titer of 6.1×10^7^ IU/ml. Polybrene was added to each T-175 flask diluted down to a final concentration of 1×. Then 1 mL of virus was added to each flask. Cells were allowed to grow over night and then the virus-containing media was aspirated and replaced with fresh virus-free media. Transduction efficiency was confirmed by analyzing GFP expression using fluorescence microscopy and flow cytometery.

Fully confluent cells were trypsinized with 1× Trypsin (Fisher) and re-suspended in conditioning media at a density of 1×10^6^ cells per 200 uL. 200 uL of cell suspension was injected into each mouse via tail vein. Blood was collected in Eppendorf tubes containing 4 µL of 20 mM EDTA via tail vein at 0.5 hour, 3 hour, 8 hour, 24 hour, 72 hour and 1 week post-MSCs injection. 10 uL blood was mixed with 100 uL 5 ug/ml coelenterazine substrate in a white, opaque 96-well plate and luminescence was detected using a BioTek microplate reader.

### Brefeldin A Treatment of MSCs and Proliferation Assay

In a 6-well plate, MSCs were incubated with brefeldin A (Sigma-Aldrich) at a final concentration of 50, 10, 5, and 1 µg/ml for 24 hours. Supernatants were collected, and then MSCs were washed 3 times using PBS. Fresh medium was replaced, and cells continued to culture up to 3 days. Supernatants were collected at different time points for subsequent human IL-6 measurements. To measure the proliferation, BFA-treated cells were reseeded in a 96-well flat bottom plate and cultured with fresh medium for 72 hours. Cell proliferation was measured using a MTT assay kit (ATCC) following the supplier’s recommended procedures.

### Statistical Analysis

In all studies batches of 3–8 mice from 2–3 independent experiments are reported. Raw pharmacokinetic data were analyzed using a 1-tailed Mann-Whitney U test for non-parametric data with the mean ± SEM shown or using a two way ANOVA with Tukey’s multiple comparison correction where pharmacokinetic parameters were calculated based on MATLAB software package models.

## Supporting Information

Figure S1 **Immunophenotyping of MSCs.** Expanded cells were CD11b−, CD45−, CD45+, and CD73+ consistent with a bone marrow MSC identity.
(TIF)

Figure S2 **Pharmacokinetics of MSC-derived IL-8 and MCP-1 after IV transplantation.** ELISA measurements of mice injected with MSCs and analyzed for human (A) MCP-1 and (B) IL-8 over time. Kinetics follow a similar trend compared to IL-6.
(TIF)
