# Correction: Pharmacokinetics of Natural and Engineered Secreted Factors Delivered by Mesenchymal Stromal Cells

**DOI:** 10.1371/journal.pone.0099813

**Published:** 2014-06-03

**Authors:** The PLOS ONE Staff

The second author’s name is spelled incorrectly. The correct author name is Ryan C. Murray. The correct citation is: Elman JS, Murray RC, Wang F, Shen K, Gao S, et al. (2014) Pharmacokinetics of Natural and Engineered Secreted Factors Delivered by Mesenchymal Stromal Cells. PLoS ONE 9(2): e89882. doi:10.1371/journal.pone.0089882
