# Supplementary figures and images for: Pharmacokinetics of Natural and Engineered Secreted Factors Delivered by Mesenchymal Stromal Cells
Source: PLoS One. 2014 Feb 21;9(2):e89882. doi: 10.1371/journal.pone.0089882 (PMC3931832; doi:10.1371/journal.pone.0089882)

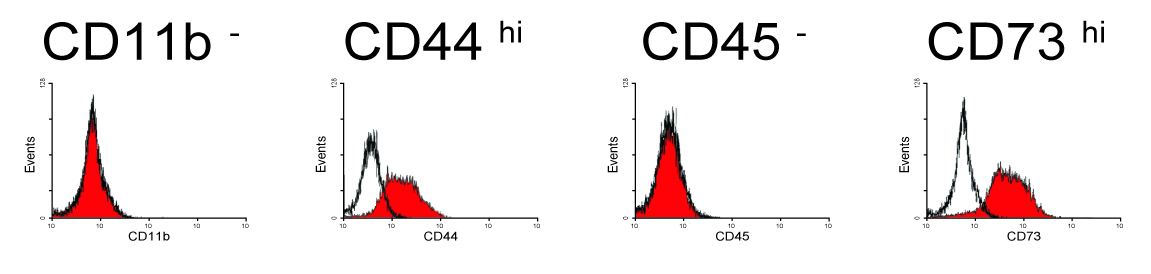

Supplement: Figure S1 — Immunophenotyping of MSCs. Expanded cells were CD11b−, CD45−, CD45+, and CD73+ consistent with a bone marrow MSC identity. (TIF) [file pone.0089882.s001.tif]

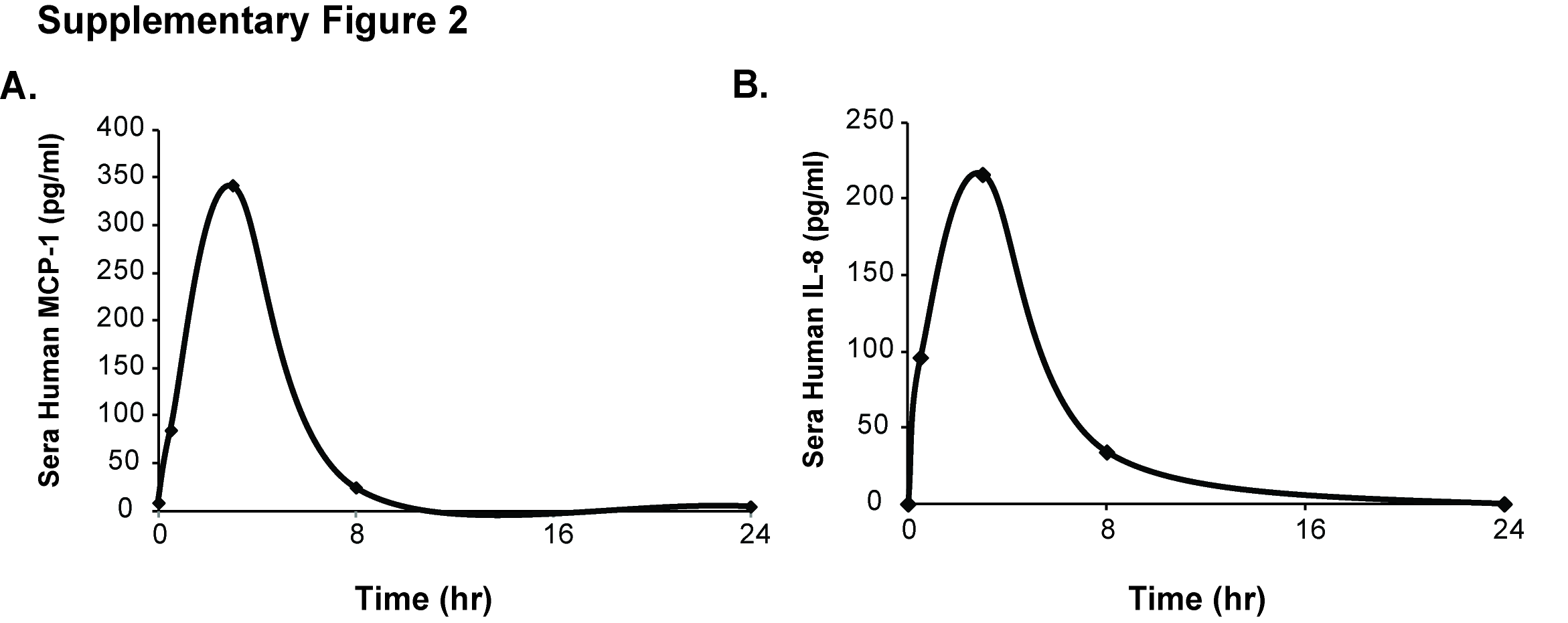

Supplement: Figure S2 — Pharmacokinetics of MSC-derived IL-8 and MCP-1 after IV transplantation. ELISA measurements of mice injected with MSCs and analyzed for human (A) MCP-1 and (B) IL-8 over time. Kinetics follow a similar trend compared to IL-6. (TIF) [file pone.0089882.s002.tif]
